# Severity-stratified gut microbiome dysbiosis and systemic neuroinflammation in acute traumatic brain injury: a metagenomic and cytokine profiling study

**DOI:** 10.3389/fmicb.2026.1845360

**Published:** 2026-08-13

**Authors:** Yiyang Wang, Yudan Zhang, Chune Li, Ruina Liu, Shuo Zhang

**Affiliations:** 1 Center for Translational Medicine, The First Affiliated Hospital of Xi'an Jiaotong University, Xi'an, China; 2 Center for Brain Science, The First Affiliated Hospital of Xi'an Jiaotong University, Xi'an, China; 3 Department of Psychiatry, The First Affiliated Hospital of Xi'an Jiaotong University, Xi'an, China

**Keywords:** butyrate, gut microbiome, gut-brain axis, metagenomics, neuroinflammation, traumatic brain injury

## Abstract

**Background:**

The gut-brain axis has increasingly been implicated in the pathophysiology of traumatic brain injury (TBI). However, few human studies have simultaneously examined gut functional metagenomics and peripheral cytokine profiles across mild to moderate-to-severe TBI, which limits our understanding of how gut health may influence recovery outcomes in TBI patients.

**Methods:**

This cross-sectional case-control investigation involved the collection of fecal and matched serum samples within 7 days post-injury from 60 mild TBI patients (MT; GCS 13–15), 45 moderate-to-severe TBI patients (MST; GCS ≤ 12), and 113 healthy controls (HC). Shotgun metagenomic sequencing examined gut microbiota. Serum IL-1β, IL-6, IL-8, and TNF-α were measured using a 4-plex Luminex test. Spearman correlation was utilized to construct a hypothesis relating cytokine levels to microbial severity-stratified abundance patterns.

**Results:**

TBI was associated with severity-dependent remodeling of the gut microbiota. Alpha diversity decreased from HC to MST (*p* < 10^−6^), and community structure varied significantly among all three groups (PERMANOVA, p = 0.001). Serum TNF-α increased in a severity-associated manner (MST vs. HC and MT, *p*_*adj*_ < 0.015). Among 332 differentially abundant species, butyrate-producing commensals fell abruptly at MT with no additional decline in MST, suggesting an apparent floor-like pattern rather than confirming a true biological floor effect. For example, *Faecalibacterium prausnitzii* dropped from 9.02% in HC to 4.29% in MT. In terms of function, metagenomic inference indicated that pathways for fermentative metabolism and short-chain fatty acid (SCFA) biosynthesis were largely suppressed, suggesting a predicted reduction in microbial SCFA production capacity. On the other hand, secondary bile acid synthesis was specifically increased in MST-dominant KOs (85%). Systemically, reduced commensals showed weak, directionally consistent correlations with pro-inflammatory cytokines (|ρ| = 0.16–0.25), although none of the species-cytokine associations survived FDR correction.

**Conclusion:**

Acute TBI is associated with severity-specific gut dysbiosis, which is accompanied by systemic neuroinflammation. The early reduction of butyrate-producing taxa in mild TBI suggests that the early post-injury period may represent a potential window for future gut-targeted intervention studies.

## Introduction

Traumatic brain injury (TBI) is a significant global public health issue. According to the most recent Global Burden of Disease (GBD) data, approximately 27 million new cases of TBI are reported annually, rendering it a significant cause of both disability and mortality (Guan et al., 2023). However, despite years of research and clinical trials focusing on the secondary injury process, no neuroprotective drugs have yet been approved by regulatory authorities (Hanscom et al., 2021b; Xiong et al., 2025).

The limitations of this translational application stem from the inherent heterogeneity of TBI patients, methodological limitations in trial design, and a lack of understanding of the subsequent injury cascade (El Baassiri et al., 2024). The pathological effects of TBI are traditionally divided into two stages: the first stage of injury, namely direct mechanical injury, and the subsequent secondary injury. The latter is triggered by a multifaceted neuroinflammatory response characterized by microglial activation, release of inflammatory cytokines (including IL-1β, IL-6, and TNF-α), and increased blood-brain barrier permeability. This second phase is an important feature that may be altered, and it plays a significant role in determining the long-term prognosis for patients (Hanscom et al., 2021b; El Baassiri et al., 2024; Mallick et al., 2025; Oyovwi and Udi, 2025). Secondary injury, a complex and multifaceted process, can last for a long time, possibly months or even years. Such injury can worsen both the neuropathological changes and the functional impairments (Hanscom et al., 2021a; Cotoia et al., 2024). Therefore, further research into the biological characteristics of secondary injury is necessary to develop novel therapeutic approaches.

The gut-brain axis has recently become a focus of research concerning central nervous system disorders. After an injury, damage-associated molecular patterns (DAMPs) and cytokines released due to the brain injury can activate the autonomic nervous system (ANS) and the hypothalamic-pituitary-adrenal (HPA) axis. Both the vagus nerve and the sympathetic stress fibers are responsible for this activation phenomenon. Therefore, this activation causes changes in the gut microbiota, which promotes the growth of harmful bacteria (Lin et al., 2025). Furthermore, this process increases the permeability of the intestinal epithelial barrier, leading to a condition commonly known as “leaking.” This increased permeability of the intestinal epithelial barrier results in a condition commonly known as “leaky gut.” (El Baassiri et al., 2024; Wu et al., 2026). Changes in the gut microbiome make it easier for pathogens and their metabolites (including the endotoxin lipopolysaccharide, LPS) to enter the systemic circulation, triggering systemic inflammatory response syndrome (SIRS). This process forms a “bidirectional damage amplification loop,” releasing numerous important inflammatory cytokines such as TNF-α, IL-1β, IL-6, and IL-8 into the serum, exacerbating inflammatory responses in the central nervous system and throughout the body (Das et al., 2011; El Baassiri et al., 2024; Albert et al., 2025a). Furthermore, preclinical studies have shown that adding butyrate-producing probiotics (such as *Clostridium butyricum*) or short-chain fatty acids (SCFAs) can directly restore the IL-22/Reg3 pathway in the intestinal barrier, thereby lowering serum levels of IL-1β, IL-6, and TNF-α, and improving neurocognitive functions such as spatial learning (Opeyemi et al., 2021; Liu et al., 2025).

However, most of the current research is confined to animal models, with a severe scarcity of human TBI cohort studies. There is a lack of (1) high-resolution analysis of gut microbiota composition and functional pathways based on metagenomics; (2) cross-omics studies integrating serum cytokine profiling; and (3) injury stratification covering both mild and moderate-to-severe subgroups. Published cohort studies mainly use 16S rRNA amplicon sequencing (Wu et al., 2026). This method identifies bacterial taxonomic composition but provides limited resolution of functional potential, such as SCFA biosynthesis capacity or genes related to LPS production (Cryan et al., 2019). Specifically, while previous human TBI microbiome studies have primarily relied on 16S rRNA amplicon sequencing in heterogeneous cohorts without concurrent systemic immune profiling (Di Battista et al., 2016; Yue et al., 2023), the present study uniquely integrates shotgun metagenomic sequencing with multiplex cytokine analysis across a continuous spectrum of TBI severity (mild to moderate-to-severe), enabling functional pathway resolution not achievable with amplicon-based approaches. Because mild and moderate-to-severe TBI represent clinically important subgroups with potentially distinct immune responses, integrated multi-omics data across these severity strata are needed. This will help us create a more comprehensive understanding of the microbiota-immune-brain axis.

To address these gaps, this study employed a case-control design. It included healthy controls (HC, *n* = 113), mild TBI patients (MT, *n* = 60), and moderate-to-severe TBI patients (MST, *n* = 45). Fecal and serum samples were collected at the same time, within seven days of the injury. Shotgun metagenomic sequencing was used to analyze microbial features and gut functional profiles. Systemic immune status was characterized using a validated 4-plex Luminex cytokine panel (detecting IL-1β, IL-6, IL-8, and TNF-α). By integrating whole-genome metagenomic sequencing with multiplex cytokine profiling, this study systematically delineates, for the first time in the acute phase (within 7 days) of human TBI, the severity-dependent synergistic patterns of gut microbiome dysbiosis and systemic inflammatory responses. It identifies differentially abundant species and key metabolic pathways and quantifies their associations with pro-inflammatory cytokine profiles, aiming to provide a human cohort-level theoretical basis for microbiome–immune-targeted interventions in both mild and moderate-to-severe TBI.

## Methods

### Recruitment of participants

This cross-sectional multi-omics case-control study enrolled a total of 218 participants aged 18–70 years, including TBI patients stratified by injury severity (mild TBI [MT], n = 60; moderate-to-severe TBI [MST], n = 45) and healthy controls (HC, n = 113). TBI severity was classified using the Glasgow Coma Scale (GCS) score assessed within 2 h post-injury: MT was defined as GCS 13–15 and MST was defined as GCS ≤ 12; this group primarily consisted of moderate TBI cases, which accounted for 88.9% of the MST cohort. All TBI patients were hospitalized within 6 h post-injury; fecal and paired serum samples were collected within 7 days after admission to capture acute gut responses prior to the potential confounding effects of prolonged hospitalization, antibiotic exposure, or dietary changes. Data were collected from the healthy control group at the time of enrollment. Age, sex, body mass index (BMI), and education level were recorded as covariates for subsequent statistical analysis. This study protocol followed the principles of the Declaration of Helsinki and was approved by the ethics committees of Xi'an Jiaotong University and the participating hospitals. All participants signed written informed consent.

### Inclusion and exclusion criteria

Inclusion criteria for the TBI cohort comprised a clinical diagnosis of closed TBI by a board-certified physician and an admission Glasgow Coma Scale (GCS) score of ≤ 15. Healthy controls recruited from the hospital's health examination center were required to be free from any history of head trauma, neurological disorders, or acute/chronic systemic diseases. The HC group, which stands for the healthy control group, was demographically matched to the TBI cohort, which refers to the traumatic brain injury cohort.

General exclusion criteria applied to all subjects included: (1) administration of antibiotics, probiotics, prebiotics, or systemic corticosteroids within 4 weeks prior to enrollment; (2) gastrointestinal infection within the preceding 3 months; (3) history of neurological or chronic psychiatric disorders; (4) pre-existing gastrointestinal diseases (e.g., inflammatory bowel disease, irritable bowel syndrome, celiac disease, colorectal cancer, or gastrointestinal surgery within the prior 3 months); (5) active autoimmune diseases or concurrent immunosuppressive therapy; (6) active malignancy, type 1 diabetes mellitus, or end-stage hepatic/renal disease; and (7) pregnancy or lactation.

Exclusion criteria for the TBI group included penetrating TBI, polytrauma of > 3 in any extracranial location, pre-existing neurological impairments, and neurosurgical intervention before biospecimen collection. Finally, subjects who could not give 0.5 g of feces or enough serum volume were eliminated from the trial.

### Multiplex cytokine measurements

In this investigation, 47 HC, 60 MT, and 45 MST serum samples were collected. The serum levels of IL-1β, IL-6, IL-8, and TNF-α were measured using a 4-plex Luminex bead-based multiplex assay system (R&D Systems, Texas, USA) on the Luminex 200 platform (Luminex, USA). Spearman correlation analysis was used to test hypotheses about microbial abundance and cytokine concentrations due to the cross-sectional study design and small sample size. Because cytokine concentrations showed right-skewed distributions, individual-level cytokine values were log10-transformed before statistical analysis. Group-wise cytokine profiles were summarized as median [interquartile range, IQR] of log10-transformed concentrations and reported together with overall and pairwise adjusted *p*-values.

### Fecal sample collection, DNA extraction, and metagenomic sequencing

Fecal samples were collected within 7 days after injury and preserved at −80 °C. Genomic DNA was obtained from feces using the QIAamp DNA Fecal Mini Kit (Qiagen, Hilden, Germany) according to manufacturer instructions. Xi'an-based Shaan Probiomicros Co., Ltd. used the BGI MGISEQ-T7 technology and paired-end 150 bp sequencing for metagenomic shotgun sequencing. Fastp (v0.23.0; parameters: length_required = 50, n_base_limit = 2) removed low-quality sequences and adapter-related impurities from raw sequencing reads. After that, Bowtie2 (v2.3.5.1) aligned host reads to GRCh38 to filter them out.

### Severity-stratified abundance pattern classification and differentially abundant species identification

Taxonomic profiling was performed using MetaPhlAn4 to describe the organization of the microbiome community and find out how common each species was. To find out how diverse the microbes were, the Shannon and Simpson indices were used. The Wilcoxon test was then used to see if the case and control groups had different diversity scores. The samples were also put into three groups: HC, MT, and MST. At first, the Kruskal–Wallis rank-sum test was used to see if there were any differences between the three groups in all of the gut microbes that had been discovered. This was followed by multiple testing corrections using the Benjamini–Hochberg method (BH-FDR; corrected q < 0.05), which showed 332 species that were significantly more or less common in different areas. Subsequently, pairwise comparisons among the three groups (HC vs. MT, HC vs. MST, MT vs. MST) were performed using the Wilcoxon rank-sum tests with Benjamini-Hochberg FDR correction (*q* < 0.05). Fifty representative differentially abundant species were selected for heatmap visualization. Data were transformed using log10 (Abundance + 10^−4^) and Z-score normalized along rows. In the heatmap, samples were arranged according to the gradient of clinical severity, and species were ordered based on statistical severity-stratified abundance patterns, with no hierarchical clustering applied to either axis to preserve the continuity of disease severity-dependent patterns in the visualization.

### Functional pathway enrichment analysis

Microbial metabolic pathways and functional modules were analyzed using HUMAnN3 (v 3.9). The GRSA workflow was employed to evaluate the overall enrichment of KEGG metabolic pathways among the three groups. For each KEGG Orthology (KO) entry, intergroup differential analysis was performed, and *p*-values were normalized into z-scores, which were then aggregated by pathway to calculate the Reporter Score. Metabolic pathways with an FDR < 0.05 and an absolute Reporter Score greater than 1.96 were identified as significantly enriched. All statistical analyses and visualizations were conducted in the R programming environment.

## Statistical analysis

The independent samples *t*-test examined continuous variables between two groups, while the chi-square test compared categorical variables. Benjamini–Hochberg (BH) was used to control FDR in multiple hypothesis tests. Microbial community composition was evaluated using vegan (2.6-10) in R. Permutational multivariate analysis of variance was used to investigate the significance of community structure differences derived using the Bray–Curtis distance matrix. Vegan also calculated alpha diversity indices. KO relative abundance was used to analyze pathway enrichment using ReporterScore (version 0.1.9) in R. Spearman's rank correlation coefficient was used to assess correlations between microbial species abundance and serum cytokine concentrations; resulting *p*-values were adjusted for multiple comparisons across all species-cytokine pairs using the Benjamini-Hochberg (BH-FDR) method, and both nominal (uncorrected) and FDR-adjusted (q) values are reported. To account for potential confounding, age group, sex, BMI, and education level were included as covariates in all PERMANOVA models using the by = “margin” argument in adonis2. All statistical studies used R (4.5.2).

## Results

**Figure 1:**
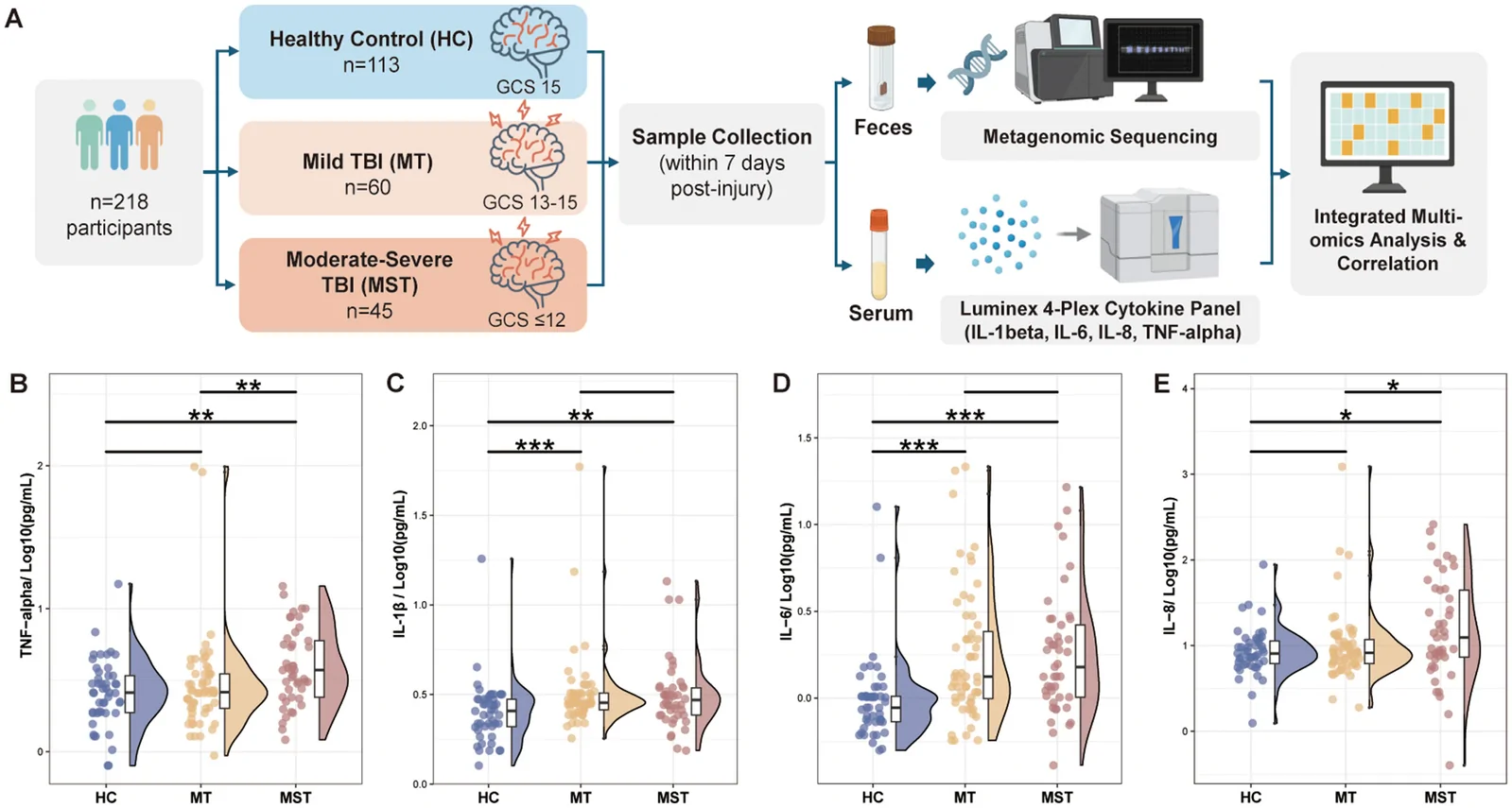
Study cohort design and acute systemic pro-inflammatory cytokine profiles stratified by TBI severity. **(A)** Flowchart of the study. HC (*n* = 113), MT (*n* = 60), and MST (*n* = 45) were enrolled; fecal and serum samples were collected within 7 days post-injury for metagenomic and cytokine analyses. **(B–E)** Boxplots showing serum levels of TNF-α, IL-1β, IL-6, and IL-8 across groups (HC, *n* = 47; MT, *n* = 60; MST, *n* = 45). Group differences were assessed by Kruskal-Wallis test followed by pairwise Wilcoxon rank-sum tests with Benjamini-Hochberg FDR correction. *p < 0.05, **p < 0.01, ***p < 0.001.

Figure 1A shows the cohort design and study flowchart. This cross-sectional study enrolled 218 people. Patients with TBI were divided into MT (GCS 13-15, *n* = 60) and MST (GCS ≤ 12, *n* = 45) groups based on GCS scores at admission. Additionally, matched healthy volunteers were included as a control HC group (n = 113). To accurately capture critical biological changes during the acute phase and avoid confounding effects from prolonged hospitalization and antibiotic treatment, fecal and corresponding peripheral serum samples were collected from patients within 7 days of injury. After nucleic acid extraction, metagenomic shotgun sequencing was performed on fecal samples to comprehensively analyze the species composition and functional metabolic capacity of the gut microbiota. A Luminex 4-plex bead-based multiplex assay was used to quantify serum IL-1β, IL-6, IL-8, and TNF-α. Finally, the aforementioned multi-omics datasets were systematically integrated using various statistical methods.

### Baseline characteristics and systemic inflammatory response patterns in TBI patients

The baseline demographic and clinical characteristics of the study cohort were shown in Table 1. Statistical analysis of the baseline data across groups revealed a balanced distribution of core demographic and physiological indicators. No significant intergroup differences were found for age grouping (*p* = 0.643), sex composition (*p* = 0.903), or body mass index (BMI, *p* = 0.217), indicating that the major confounding factors were comparable among the groups. Regarding other characteristics, a statistically significant difference in years of education was observed among the three groups (*p* < 0.001). Compared to the HC group (median: 6.0 years), the TBI patient groups exhibited higher median years of education (9.0 years in the MT group; 10.0 years in the MST group). In terms of clinical severity, consistent with the inclusion criteria for each group, the admission Glasgow Coma Scale (GCS) score showed significant stratified differences among the groups (*p* < 0.001). The median score significantly decreased from 15 in the HC group and 14 in the MT group to 11 in the MST group, reflecting an increasing trend in the severity of acute central nervous system injury across the cohort.

**Table 1:** Demographics and clinical characteristics of study populations.

Variable	HC (*n* = 113)	MT (*n* = 60)	MST (*n* = 45)	*P-*value
Age, *n* (%)				
< 30	10 (8.8%)	7 (11.7%)	6 (13.3%)	0.643
30-50	43 (38.1%)	25 (41.7%)	21 (46.7%)	
15.6-7.2,-1.3498pt >50	60 (53.1%)	28 (46.7%)	18 (40.0%)	
Gender, *n* (%)				
Male	58 (51.3%)	32 (53.3%)	22 (48.9%)	0.903
Female	55 (48.7%)	28 (46.7%)	23 (51.1%)	
**BMI (kg/m^2^), median (IQR)**	23.24 [21.47-25.04]	22.03 [20.26-24.54]	21.48 [19.46-28.29]	0.217
**Education (years), median (IQR)**	6.00 [0.00-9.00]	9.00 [9.00-12.25]	10.00 [5.00-13.00]	< 0.001
**GCS, median (IQR)**	15.00 [15.00-15.00]	14.00 [14.00-15.00]	11.00 [10.00-11.00]	< 0.001

^*^GCS scores in the HC group represent the baseline neurological status of healthy individuals and are presented for completeness of comparison; they are not used as a clinical severity indicator in this population.

^*^Data are represented as median [interquartile range, IQR] for quantitative variables and frequency for categorical variables. Statistical differences were determined using the chi-square test for categorical variables and the Kruskal-Wallis H test for quantitative variables.

We systematically compared serum inflammatory cytokine levels among the three groups using a Luminex bead-based multiplex assay platform (Figures 1B–E). Kruskal-Wallis test results showed significant differences in IL-6 (*p*_*adj*_ < 0.001), TNF-α (*p*_*adj*_ = 0.002), and IL-1β (*p*_*adj*_ = 0.002) levels among the three groups. However, IL-8 did not reach the corrected significance threshold (*p*_*adj*_ = 0.056). Pairwise comparisons showed that IL-6 and IL-1β were significantly elevated in both MT and MST groups compared with HC, whereas no significant difference was observed between the MT and MST groups. The MST group exhibited a substantial elevation in TNF-α levels relative to both the HC and MT groups (*p*_*adj*_ = 0.003 and 0.008, respectively), indicating a severity-dependent increase. IL-8 showed a more variable profile: although the overall adjusted *p*-value did not reach the conventional significance threshold, IL-8 levels were significantly higher in the MST group than in the HC group in pairwise comparison (*p*_*ad*_*j* = 0.027), with substantial within-group variability in the MST group. To provide a more detailed cytokine profile, group-wise distributions of TNF-α, IL-1β, IL-6, and IL-8 are summarized in Supplementary Table 1 as median [IQR] of log10-transformed concentrations. Overall, IL-6 and IL-1β were elevated in both TBI groups compared with HC, whereas TNF-α showed the clearest severity-associated pattern, with higher levels in MST than in both HC and MT.

### Gut microbial community structure changes with TBI severity

**Figure 2:**
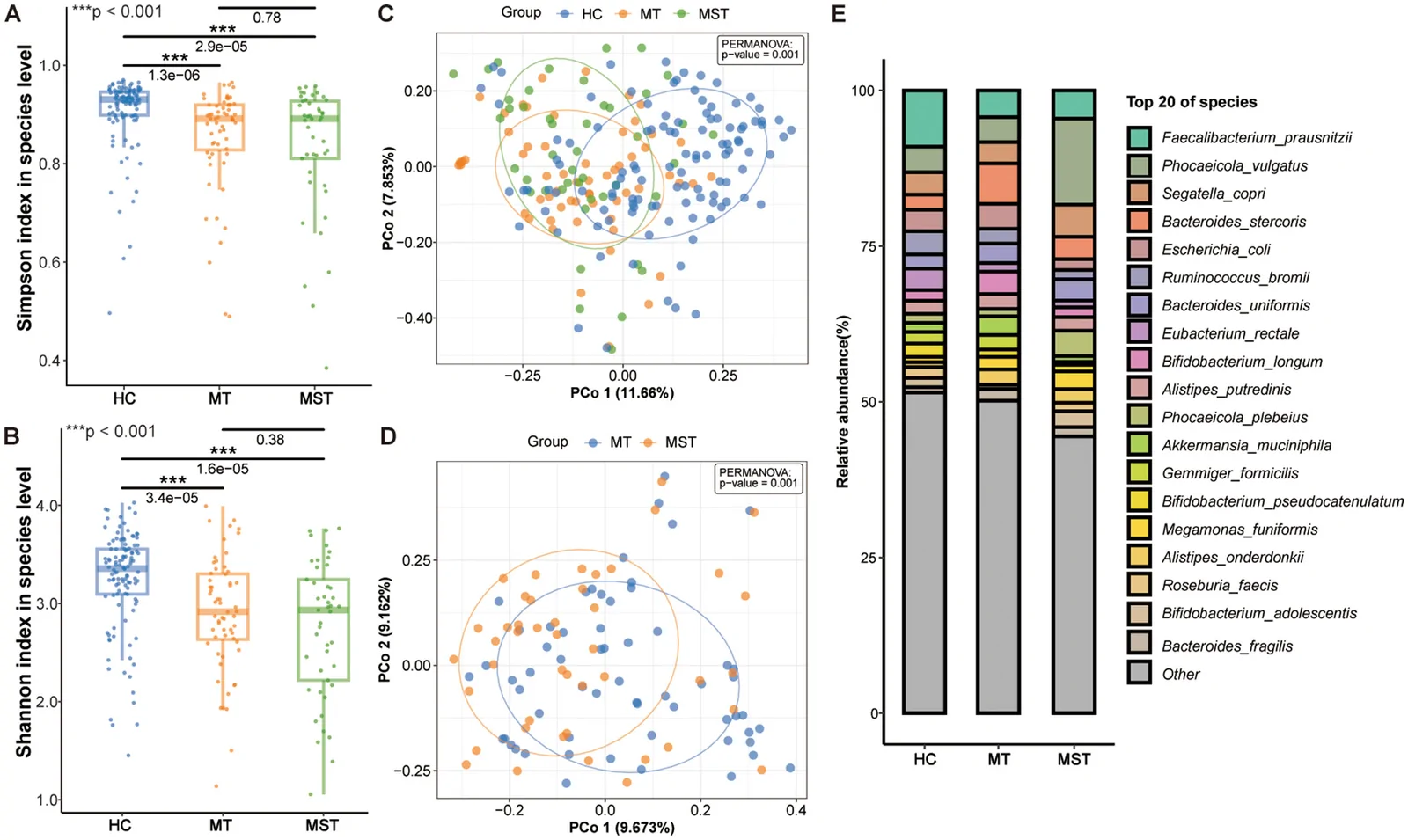
Gut microbiota community structure across TBI severity groups (HC, *n* = 113; MT, *n* = 60; MST, *n* = 45). **(A, B)** Alpha diversity measured by Simpson **(A)** and Shannon **(B)** indices; compared by Wilcoxon rank-sum tests with Benjamini-Hochberg FDR correction. PCoA plots based on Bray-Curtis dissimilarity among three groups **(C)** and between MT and MST **(D);** group separation assessed by PERMANOVA (999 permutations) with age, sex, BMI, and education as covariates. **(E)** Stacked bar charts of dominant species-level taxa; each bar represents group mean. *p < 0.05, **p < 0.01, ***p < 0.001.

TBI was associated with broad alterations in the gut microbiota. The severity of the injury also affected these alterations. Shannon and Simpson indices indicate a decreasing trend in alpha diversity, with HC > MT > MST (Kruskal–Wallis test, p < 10^−6^) (Figures 2A, B). The MST cohort had a higher sample dispersion, suggesting that severe injury patients' gut microbiota were more heterogeneous. Beta diversity analysis, shown in Figures 2C, D, using Principal Coordinates Analysis (PCoA) of Bray-Curtis distance, showed clear differences in microbial community structures among the three groups and the MT and MST groups (PERMANOVA, *p* = 0.001). These findings indicate that TBI is associated with broad changes in microbial abundance and community organization. After adjusting for age group, sex, BMI, and education level as covariates (adonis2, by = “margin”), the injury group effect remained highly significant (*R*^2^ = 0.042, F = 4.79, *p* = 0.0001). Age group showed a small but statistically significant independent contribution to community structure (R^2^ = 0.007, p = 0.030), approximately six-fold smaller than the injury group effect, while education level showed a comparably small but significant independent association (R^2^ = 0.012, F = 2.77, *p* = 0.0001). These results suggest that the observed microbiome differences across groups were not fully explained by the age or educational imbalances noted in Table 1.

In this study, bacterial species were operationally defined as “commensals” or “opportunistic pathogens” based on converging evidence in the literature, including three criteria: (1) confirmed production of immunoregulatory metabolites (e.g., butyrate and other SCFAs); (2) reported association with anti-inflammatory or pro-inflammatory outcomes in human or animal studies; and (3) known patterns of enrichment or depletion related to gut barrier disruption or systemic inflammatory conditions. This operational definition is in line with the accepted definition of “pathobiont,” which refers to resident commensal bacteria that acquire pathogenic potential only under certain host or environmental conditions, rather than being fundamentally pathogenic (Chow and Mazmanian, 2010). *Faecalibacterium prausnitzii, Ruminococcus bromii*, and members of the *Bifidobacterium* genus were classified as commensals because of their well-known anti-inflammatory, barrier-protective, and short-chain fatty acid-producing properties (Sokol et al., 2008; Lopez-Siles et al., 2017; Cryan et al., 2019). Conversely, *Phocaeicola vulgatus* and *Phocaeicola plebeius* were identified as taxa with context-dependent pro-inflammatory potential, rather than classical opportunistic pathogens, due to their known association with pro-inflammatory outcomes and their increased prevalence in conditions of gut barrier dysfunction and severe disease (Albert et al., 2025a). This classification indicates that pathogenicity is context dependent and not a definitive taxonomic category, as many species are also found in healthy individuals. We note that there are currently no standardized criteria across the field to classify gut bacteria along the commensal-pathobiont spectrum (Jochum and Stecher, 2020); to maximize transparency and reproducibility of this operational classification, we explicitly enumerate the three criteria above. This operational classification was applied only to the representative taxa mentioned in this study and not meant as an exhaustive pathogenicity assignment for all 332 differentially abundant species found.

As the severity of injury increases, the abundance of commensal bacteria with anti-inflammatory properties, such as *Faecalibacterium prausnitzii, Ruminococcus bromii*, and the *Bifidobacterium* genus, decreases, while the abundance of pathobiont-like taxa with context-dependent pro-inflammatory potential, such as *Phocaeicola vulgatus* and *Phocaeicola plebeius*, increased. These changes suggest a shift toward a more pro-inflammatory microbial configuration after TBI. Furthermore, *Akkermansia muciniphila, Segatella copri*, and *Bacteroides stercoris* were found in all three groups. However, the proportions of these microorganisms varied between the groups, suggesting that TBI facilitated a systemic reshaping of the overall microbial structure rather than isolated changes in specific species (Figure 2E). Particularly, the prevalence of *Faecalibacterium prausnitzii* was significantly reduced among the TBI groups.

### Clustering of gut microbiota severity-stratified abundance patterns of gut microbiota in TBI

**Figure 3:**
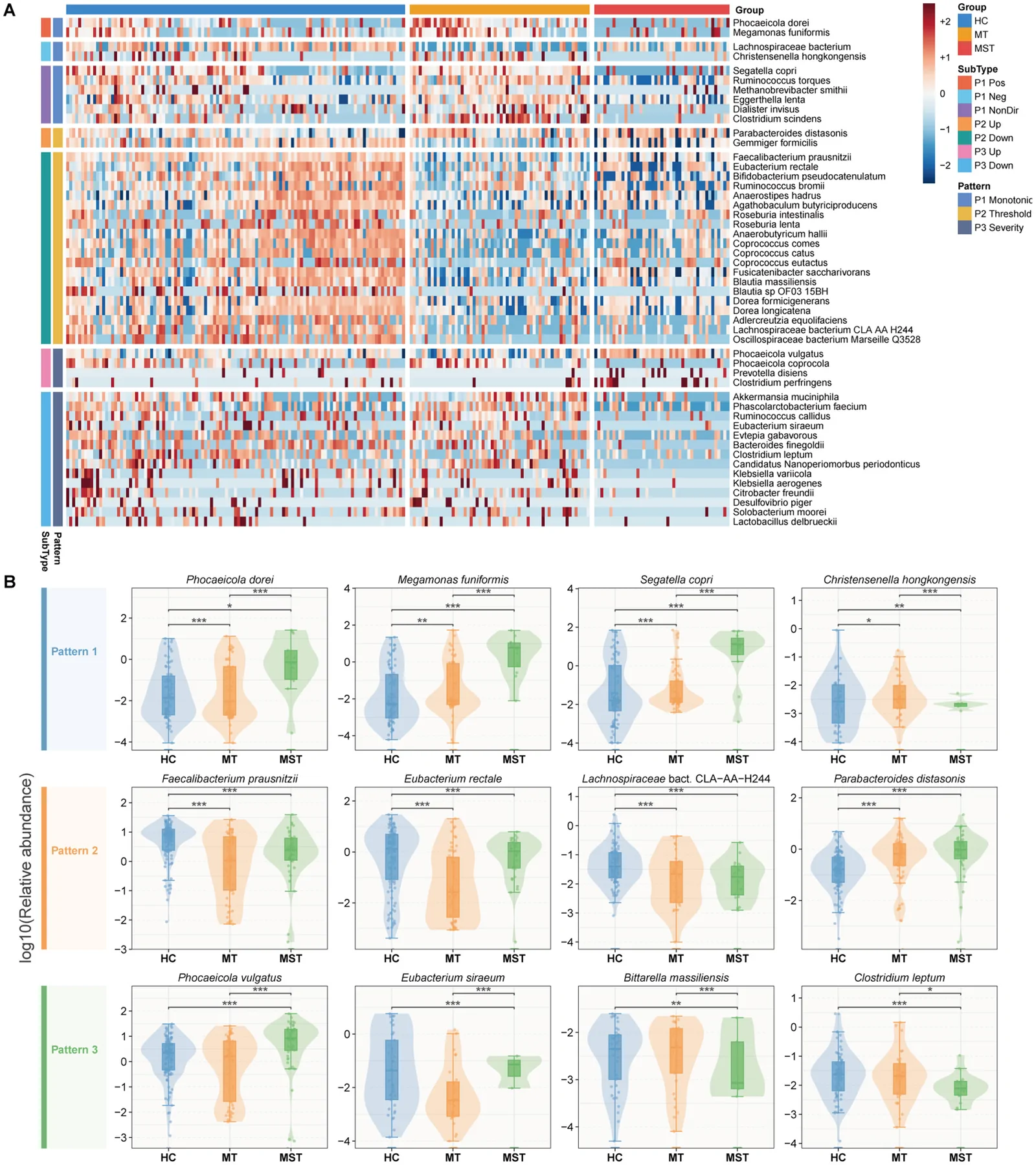
Severity-associated alterations clustering of gut microbiota in TBI (HC, n = 113; MT, n = 60; MST, n = 45). **(A)** Heatmap of 50 representative differentially abundant species grouped into three patterns: Monotonic (10 species), Threshold (22 species), and Severity (18 species). Abundance values are log_10_-transformed and Z-score-normalized by row; columns ordered by clinical severity without hierarchical clustering. **(B)** Boxplots of representative species from each pattern across groups. Boxes indicate the median and interquartile range; whiskers extend to the most extreme non-outlier values. Compared by Wilcoxon rank-sum tests with Benjamini-Hochberg FDR correction. *p < 0.05, **p < 0.01, ***p < 0.001.

To systematically elucidate the severity-stratified abundance patterns of gut microbiota following TBI, this study categorized significantly different species into three severity-stratified abundance patterns based on the statistical characteristics of their abundance changes across the HC, MT, and MST groups (Figure 3A).

Pattern 2 (threshold pattern) consists of 69 species, making it the most species-rich and functionally important core transition pattern. Its most prominent feature is the fluctuation in the number of related species between the HC and MT groups, which can increase or decrease. Subsequently, the abundances in the MT and MST groups remained relatively stable, showing a clear “threshold shift” rather than a gradual change depending on the severity of the injury. Most species in this pattern (P2_Down) experience a population decline after injury. The species *Faecalibacterium prausnitzii* varied the most in this pattern and decreased in average relative abundance. It dropped 52 from 9.02% in the HC group to 4.29% in the MT group. The bacterial abundance observed in the MST group (4.51%) was similar to that in the MT group. This pattern suggests an apparent floor-like stabilization after mild TBI; however, it should not be interpreted as definitive evidence of a true biological floor effect. Alternative explanations include early depletion before sample collection, limited sensitivity to detect further reductions at low relative abundance, compositional constraints of relative abundance data, or distinct ecological restructuring processes in MT and MST patients. Other taxa, such as *Eubacterium rectale* (HC 3.48%, MT 1.38%, MST 1.09%), *Ruminococcus bromii* (HC 3.73%, MT 2.37%, MST 1.46%), *Bifidobacterium pseudocatenulatum* (HC 2.12%, MT 1.19%, MST 1.05%), *Anaerostipes hadrus* (HC 1.45%, MT 0.29%, MST 0.61%), and *Anaerobutyricum hallii* (HC 0.89%, MT 0.17%, MST 0.21%), all showed a very consistent threshold-like decline pattern (Figure 3B). Phylogenetically, these declining species are almost entirely confined to the Lachnospiraceae and Ruminococcaceae families within the Firmicutes phylum, which are significant producers of gut short-chain fatty acids, especially butyrate (Bai et al., 2020; Opeyemi et al., 2021). On the other hand, several bacterial species (P2_Up) showed threshold-like increases after injury. *Parabacteroides distasonis* showed a monotonic increase across groups (HC 0.42%, MT 1.57%, MST 1.82%).

Pattern 1 (monotonic pattern) included 31 species, and their abundances showed a monotonic linear change among the HC, MT, and MST groups. Specifically, the abundances of *Megamonas funiformis* and *Phocaeicola dorei* showed a monotonic increasing trend, while the abundance of *Christensenella hongkongensis* showed a monotonic decreasing trend. These results suggest the existence of a persistent positive or negative correlation between some bacterial communities and the degree of injury.

Pattern 3 (severity pattern) included 62 species. No significant difference was observed between the MT and HC groups, but a significant change was observed in the MST group compared to both groups. For example, the average abundance of *Phocaeicola vulgatus* and *Eubacterium siraeum* in the MST group reached 13.82%, significantly higher than the 4.13% in the HC group and 4.02% in the MT group, demonstrating a typical characteristic of this pattern. This finding reveals a microecological response specific to severity, which is either activated or suppressed only when a threshold of moderate-to-severe injury is surpassed.

### Functional pathway enrichment analysis of the gut microbiome

**Figure 4:**
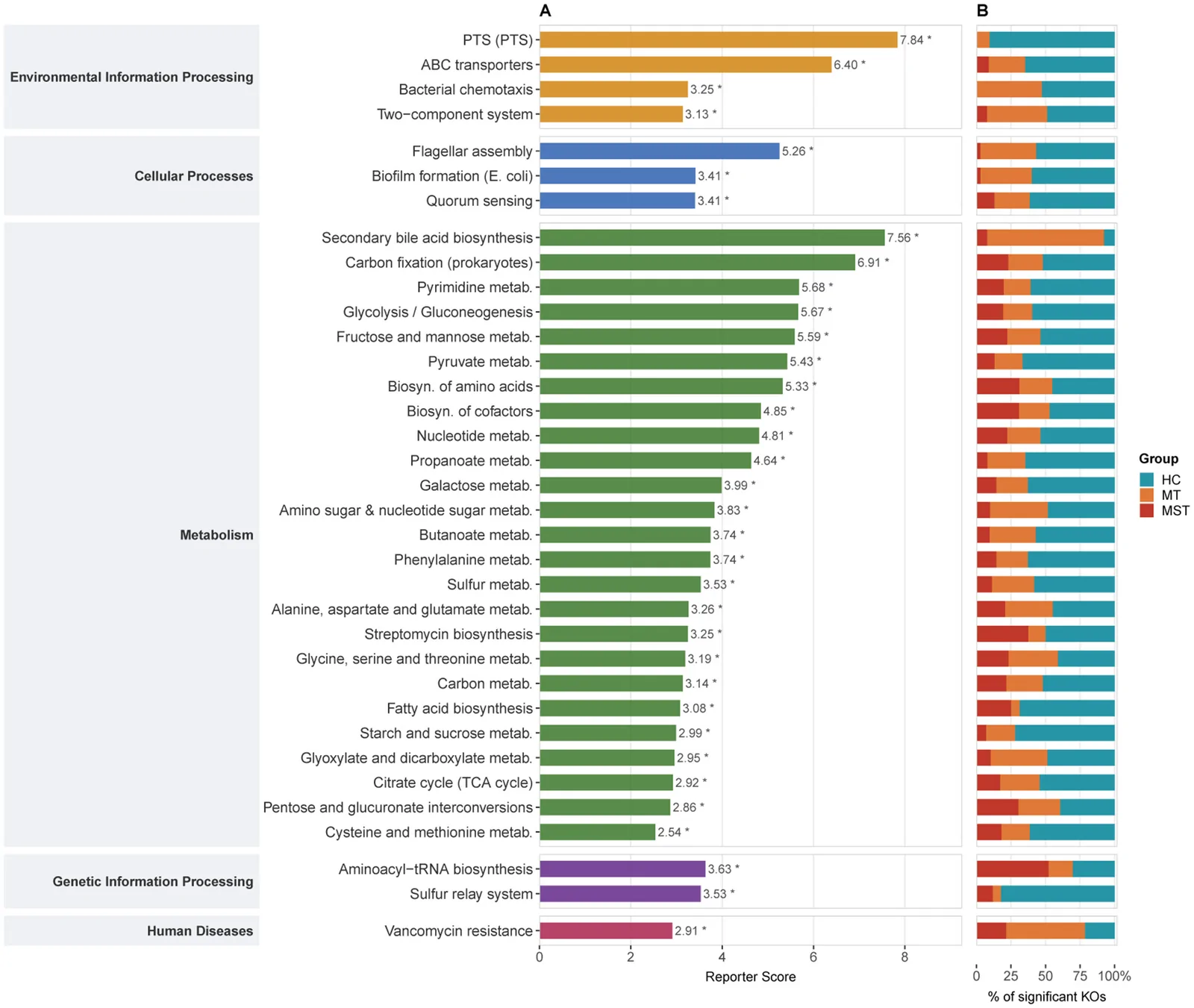
Functional pathway enrichment of the gut microbiome (HC, *n* = 113; MT, *n* = 60; MST, *n* = 45). **(A)** Reporter Score values for 35 significantly enriched KEGG pathways (FDR < 0.05, |RS| > 1.96), colored by functional module. Reporter Scores were derived from KO-level intergroup differential analysis using the GRSA workflow in HUMAnN3. **(B)** Proportion of significantly altered KOs with highest mean abundance in each group, shown overall and within selected representative pathways.

The Reporter Score analysis revealed a total of 35 KEGG pathways that were significantly enriched (FDR < 0.05), distributed across five primary functional modules (Figures 4A, B). Two major functional patterns emerged from the pathway enrichment analysis. First, most enriched pathways suggested a broad reduction in core microbial metabolic processes after TBI. Among the 1,912 significantly differential KOs, 55.5% displayed the highest average abundance within the HC group. The second finding is that the TBI group selectively enriched a few pathways, which indicates that selected functional pathways were enriched in the TBI groups, suggesting injury-associated functional reorganization rather than a uniform loss of microbial functions.

In the areas of microbial nutrition uptake and central carbon metabolism, the most substantial functional deficits were seen. The phosphotransferase system (PTS) (RS = 7.84) and ABC transporters (RS = 6.40), both of which are key bacterial nutrient absorption systems, were among the pathways that were considerably enriched. These systems are central to bacterial nutrient uptake and metabolic activity. More specifically, the prevalence of differentially abundant KOs was highest in the HC group at 91% and 65%, respectively. A similar HC dominance trend was observed in fermentation and energy production metabolic pathways, including pyruvate metabolism (67% of HC), propionic acid metabolism (65% of HC), and glycolysis/gluconeogenesis (60% of HC). Taken together, these pathway-level findings indicate that HC-dominant functional enrichment spans the majority of core metabolic processes, with TBI-associated shifts concentrated in fermentation, energy production, and nutrient uptake pathways. Not only were KO patterns seen in the pathways related to flagellar assembly (RS = 5.26), but also in the pathways related to biofilm formation (RS = 3.41) and quorum sensing (RS = 3.41).

Against this predominantly HC-dominant background, two pathways showed MST-dominant KO patterns. In contrast to the HC-dominant pattern observed in other pathways, 11 of the 13 differentially abundant KOs (85%) exhibited their peak abundance within the MST group for the predicted biosynthesis of secondary bile acids (RS = 7.56), based on KO abundance patterns inferred from metagenomics. MST dominance was evident in the vancomycin resistance pathway (RS = 2.91), with 57% of the observed differentially abundant KOs being ascribed to this specific group.

In the MT group, aminoacyl-tRNA biosynthesis was the only major pathway (52%), whereas a similar functional enrichment pattern was not observed in the MST group. This suggests that mild brain injury is associated with specific and unique disruptions to microbial translation mechanisms and that these disruptions do not increase linearly with the severity of the injury.

Overall, the functional modules associated with human diseases were the only ones with a higher proportion of major KO features in the MST group than in the HC group (57% vs. 21%). This suggests that MST-associated KO features were more frequently represented in KEGG modules related to human disease pathways, although the clinical relevance of these predicted functions requires further validation.

### Spearman correlation between gut microbiota severity-stratified abundance species and serum cytokines

**Figure 5:**
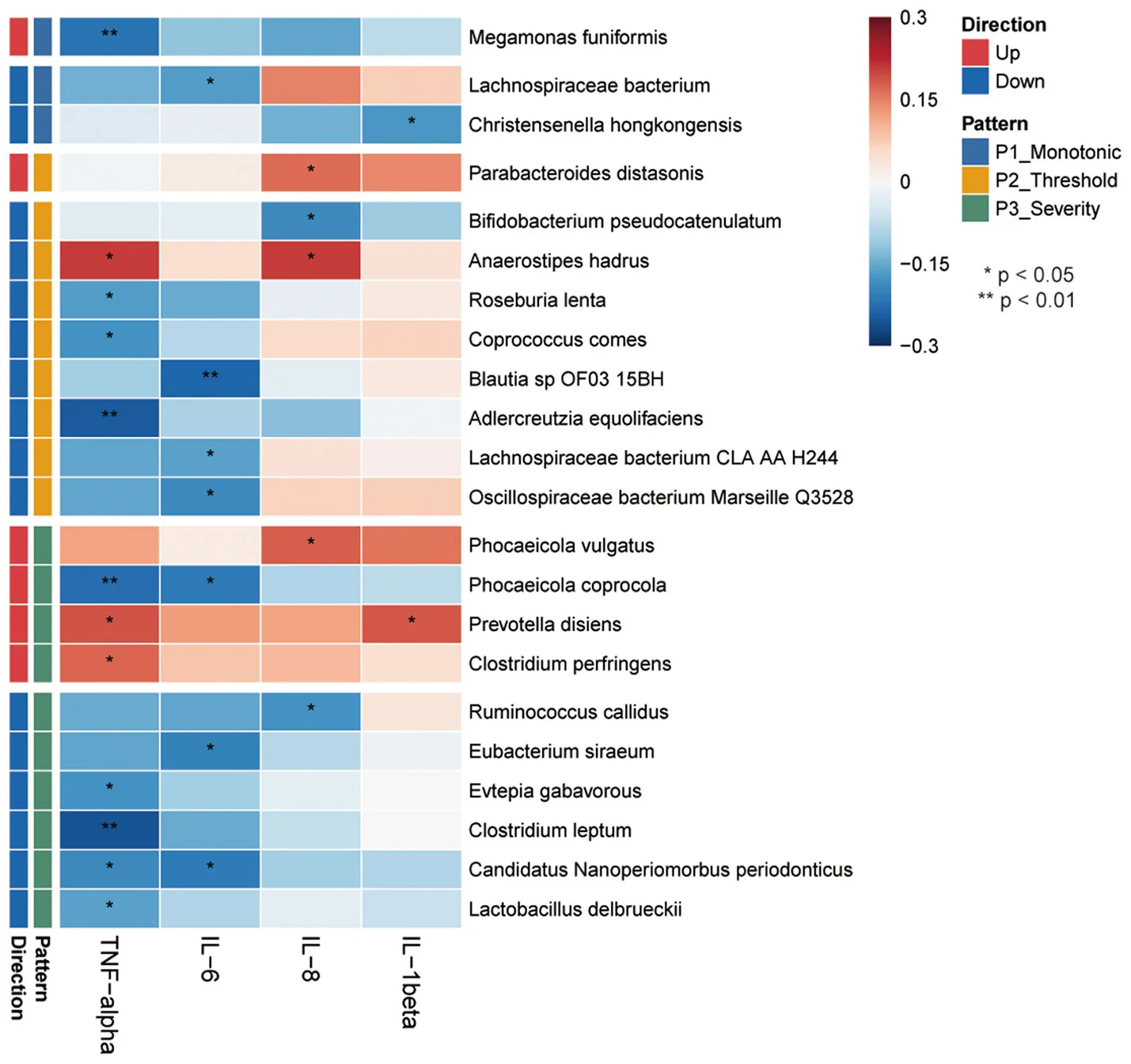
Spearman correlations between gut microbiota severity-stratified abundance species and serum cytokines (HC, *n* = 47; MT, *n* = 60; MST, *n* = 45). Species with uncorrected *p* < 0.05 for at least one cytokine are shown. Significance symbols reflect uncorrected p-values (*p < 0.05; **p < 0.01; ***p < 0.001); no associations survived Benjamini-Hochberg FDR correction (minimum *q* = 0.19). All correlations are exploratory and hypothesis-generating.

To explore the potential link between changes in the gut microbiota associated with the severity dependence of TBI and systemic neuroinflammation, this study systematically analyzed Spearman correlations between the transition patterns of three gut microbiota and serum levels of TNF-α, IL-1β, IL-6, and IL-8. Using a screening criterion of at least one cytokine with an original *p* < 0.05, a total of 20 species were included for presentation (Figure 5). Overall, the absolute values of the correlation coefficients were weak to moderate (|ρ| = 0.16–0.25). After Benjamini-Hochberg correction, none of the associations reached the conventional threshold of q < 0.05 (minimum q = 0.19); we therefore present and interpret these correlations as exploratory and hypothesis-generating rather than as confirmed, statistically robust associations.

A notable pattern was directional consistency. Species that decrease with increasing TBI severity (P2_Down and P3_Down) are almost always negatively correlated with inflammatory cytokines, while species that increase with increasing severity (P3_Up) are often positively correlated with cytokines. This consistency of directionality across many independent species-cytokine pairs could lend some plausibility to these associations at a general pattern level, but it is not a substitute for statistical significance in any individual comparison, none of which survived correction for multiple testing. Specifically, in the P3 severity category with reduced bacterial flora, several bacterial species showed nominally significant (uncorrected p < 0.05) negative correlations with both TNF-α and IL-6, constituting the most notable signals in this study. These species include *Clostridium leptum* (ρ = −0.253, q = 0.19) and *Candidatus Nanoperiomorbus periodonticus* (IL-6: ρ = −0.205, q = 0.20; TNF-α: ρ = −0.191, q = 0.26). In the P2 threshold category with reduced bacterial flora, short-chain fatty acid-producing bacteria also showed similar negative correlations with TNF-α or IL-6, including *Adlercreutzia equolifaciens* (ρ = −0.247, q = 0.19) and *Blautia sp. OF03-15BH* (ρ = −0.236, q = 0.20). On the other hand, bacterial species that increased in proportion to disease severity showed the opposite relationship. *Prevotella disiens* showed a positive correlation with both TNF-α and IL-1β (ρ = 0.19 and 0.18, respectively; both q > 0.25), while *Clostridium perfringens* showed a positive correlation with TNF-α (ρ = 0.17, q = 0.29). None of these positively-directed associations remained significant after FDR correction either. Taken together, these nominal, direction-consistent associations are consistent with a potential link between changes in microbial abundance and inflammatory marker levels, though they do not, on their own, statistically confirm such an association.

## Discussion

This study identified three distinct severity-stratified abundance patterns of gut microbiota remodeling after TBI, each characterized by different changes across the HC, MT, and MST groups. Among these, Pattern 2 (threshold pattern) was particularly notable because butyrate-producing bacteria represented by *Faecalibacterium prausnitzii* showed a substantial early reduction at the mild TBI stage, with little additional decrease in the MST group (HC 9.02% → MT 4.29% → MST 4.51%), rather than showing a linear decline along the injury severity gradient. We interpret this as a descriptive floor-like pattern rather than definitive evidence of a biological floor effect. Because samples were collected once within 7 days post-injury, early depletion before sampling, detection limits at low abundance, and injury-stage-specific ecological restructuring may also explain the apparent stabilization between MT and MST. From a biological perspective, butyrate has been reported to mitigate intestinal mucosal inflammation by inhibiting NF-κB transcriptional activity and upregulating PPARγ–a pathway exploited by *F. prausnitzii* to suppress pro-inflammatory cytokine release, thereby exerting broad anti-inflammatory effects (Lopez-Siles et al., 2017; Taraskina et al., 2022). Consistent with this functional loss, the threshold-like decrease was also consistently observed in other butyrate producers, including *Eubacterium rectale, Anaerostipes hadrus*, and *Anaerobutyricum hallii*. Phylogenetically, these species belong to the Lachnospiraceae and Ruminococcaceae families, which are the primary intestinal producers of SCFAs, especially butyrate. In contrast, Pattern 3 suggested an additional severity-associated microbial pattern: species such as *Phocaeicola vulgatus* (mean 13.82% in the MST group) were significantly enriched exclusively during the moderate-to-severe TBI stage, with no difference from healthy controls in the mild group. This microecological shift, which manifests only beyond the threshold of moderate-to-severe injury, is consistent with the possibility that more severe trauma-related physiological stress, including autonomic and HPA-axis activation, may contribute to gut microbial responses distinct from those observed in milder injury states (Albert et al., 2025b). Together, these findings are consistent with previous evidence suggesting that TBI-associated gut dysbiosis is multifaceted and may involve enrichment of pathobiont-like taxa associated with systemic inflammation (Wu et al., 2026). Furthermore, this pro-inflammatory remodeling parallels the dysbiosis documented in other acute stress conditions such as sepsis and intracranial surgery, implying that diverse forms of acute systemic stress may converge on common mechanisms associated with gut microbial imbalance (Tan et al., 2011; Zhang et al., 2024).

Functional pathway analysis revealed that the alterations in the gut microbiota following TBI extend far beyond the rearrangement of species composition but are accompanied by functional metabolic remodeling. Pathways related to fermentation and energy metabolism all exhibited highly HC-dominant enrichment, consistent with a metagenomically inferred pattern in which the predicted SCFA biosynthesis potential is progressively suppressed with increasing TBI severity. Consistent with this, preclinical evidence demonstrates that microbial production of short-chain fatty acids—particularly butyrate—attenuates long-term neurological impairment following TBI, providing mechanistic support for the functional pathway losses observed in the present study (Xiong et al., 2025). In contrast, the secondary bile acid biosynthesis pathway (Reporter Score = 7.56) showed a distinct pattern. Specifically, 85% of the differentially abundant KO terms were most common in the MST group, making it the only “MST-dominant” functional pathway identified in this study. Notably, this biosynthesis pathway co-enriched with vancomycin resistance pathways in the same group, suggesting that moderate-to-severe TBI may be associated with the expansion of microbial subpopulations with predicted pro-inflammatory metabolic potential (Mahajan et al., 2023; Benameur et al., 2025). Mechanistically, secondary bile acids modulate immune responses, oxidative stress, and blood-brain barrier integrity through receptors like FXR and TGR5. Under dysbiotic conditions, disruption of this signaling axis may contribute to microglial activation and promote the release of pro-inflammatory cytokines within the central nervous system, while also compromising gut barrier integrity and amplifying systemic inflammation (Albert et al., 2025a; Butcher and Arthur, 2025). Therefore, severe TBI was associated with a two-part predicted gut metabolic signature: an inferred decline in SCFA-related protective capacity and a metagenomically predicted enrichment of secondary bile acid biosynthesis pathways. If confirmed by direct metabolite measurement in future studies, this pattern would support the hypothesis of a self-reinforcing inflammatory loop along the gut-brain axis.

Cytokine data offer a preliminary, hypothesis-generating link to this functional integration framework. The P2_Down and P3_Down groups, characterized by reduced symbiotic bacteria, tend to show negative correlations with inflammatory cytokines (TNF-α and IL-6), while the injury-enriched taxa (P3_Up group, including *Clostridium perfringens*) tended to show positive correlations. However, as outlined in the Results, the overall correlation coefficients were weak to moderate (|ρ| = 0.16–0.25), and none of the species-cytokine comparisons passed the Benjamini-Hochberg correction for multiple testing (minimum q = 0.19). The directional consistency is indicative rather than conclusive proof of biological significance. One possible explanation for the directional trend observed is that SCFAs regulate T-cell differentiation and neuroinflammation through G-protein-coupled receptor activation and histone deacetylase inhibition (Celorrio et al., 2024). In parallel, TNF-α showed the clearest severity-associated increase among the measured cytokines, with higher levels in the MST group than in both the HC and MT groups. This pattern was directionally concordant with the enrichment of Pattern 3 taxa in MST, but it should be interpreted as cross-sectional concordance rather than evidence of a temporal or causal relationship. One possible theory is that an enrichment of pathobiont-like taxa may contribute to systemic inflammatory activation by microbial product-mediated pathways, such as LPS-TLR4-NF-κB signaling; however, this pathway was not directly assessed in the present study. These results should therefore be regarded as hypothesis-generating and require validation through direct measurements of microbial products, inflammatory signaling pathways, and longitudinal cytokine dynamics (Berezow and Darveau, 2011; Vogt et al., 2017).

Several non-mutually exclusive mechanisms may link the observed gut microbiome alterations with systemic and neuroinflammatory responses after TBI. First, TBI-induced intestinal barrier disruption (“leaky gut”) may promote the systemic transfer of lipopolysaccharides (LPS) and other microbial products from pathobiont-like taxa (Sundman et al., 2017; Albert et al., 2025a). Pathogen-associated molecular patterns (PAMPs) can activate the TLR4/NF-κB signaling cascade in peripheral immune cells and brain-resident microglia, thereby promoting the production of pro-inflammatory cytokines such as TNF-α, IL-1β, and IL-6 (Tan et al., 2011; Filidou et al., 2024). Second, the marked reduction in butyrate-producing taxa suggests a reduced microbial capacity for SCFA production, which may weaken SCFA-mediated immunoregulatory effects (Hayakawa et al., 2011; Urban et al., 2020). SCFAs are reported to maintain epithelial integrity, enhance Treg differentiation and suppress pro-inflammatory microglia phenotypes via G-protein-coupled receptor (GPCR, e.g., FFAR2/3) signaling and epigenetic regulation of histone deacetylases (HDACs) (Feighery et al., 2008; Hazeldine et al., 2015; Mahajan et al., 2023). Third, the convergence of systemic inflammation and altered microbial metabolite profiles may interact with TBI-induced blood-brain barrier (BBB) disruption (Tan et al., 2011; Zhang et al., 2024). Such barrier disruption may facilitate the trafficking of peripheral immune cells, including monocytes and T lymphocytes, into the brain parenchyma, thereby further amplifying microglia-mediated neuroinflammation (Chen et al., 2023; Yu et al., 2024). Fourth, disruption of microbial bile acid metabolism may also impair neuroimmune homeostasis and barrier function through reduced activation of the farnesoid X receptor (FXR) and TGR5 signaling pathways (Hou et al., 2022; Albert et al., 2025a). Finally, although these proposed pathways are supported by the literature, they were not directly tested in the present investigation. These pathways should therefore be viewed as biologically plausible mechanistic hypotheses that require validation in future longitudinal multi-omics studies integrating metagenomics, metabolomics, inflammatory signaling assays, and experimental models.

A potential translational implication of these findings is that early post-injury microbiome changes may represent a window for future gut-targeted intervention studies. Butyrate-producing bacteria showed a substantial reduction already in the mild TBI group. This observation suggests that future intervention studies may need to consider earlier post-injury time points rather than focusing exclusively on later or more severe disease stages. A study on mild TBI patients revealed a significant reduction in *Hungatella hathewayi*—a butyrate-producing bacterium—in the cognitive impairment group (Du et al., 2026). The same study demonstrated in an animal model that restoring *H. hathewayi* abundance through microbial transplantation led to improvements in spatial memory, decreased TNF-α and IL-6 levels in the brain, and an increased proportion of M2 microglia. These findings suggest that early microbiota-targeted intervention may have neuroprotective potential, although this remains to be tested prospectively in human TBI cohorts. While our findings are observational and the metabolic pathways are inferred from metagenomic data rather than experimentally validated, we propose the following testable predictions that may guide future hypothesis-driven investigations: (1) Early oral or enteral supplementation with SCFAs or specific probiotic formulations enriched in butyrate-producing bacteria might represent a potential intervention, though this requires direct experimental confirmation; (2) The predicted abundance profiles of SCFA synthesis pathways and secondary bile acid pathways could serve as candidate functional biomarkers for prospectively assessing the efficacy of gut-targeted interventions, provided that their predictive value is validated in independent cohorts; (3) A multi-cytokine panel including TNF-α and IL-6 may be explored as part of future composite biomarker models, but its clinical utility for acute-phase risk stratification requires prospective validation. Furthermore, follow-up data from chronic TBI patients show that abnormal changes in gut microbiota can persist for years, along with chronic inflammation, suggesting that the potential window for gut-targeted intervention may extend beyond the acute phase and should be evaluated in long-term follow-up studies (Milleville et al., 2020; Pyles et al., 2024).

While this study presents new insights into the microbiota-gut-brain axis following TBI, certain methodological considerations offer valuable directions for future research. First, the cross-sectional design and single sampling time point within 7 days post-injury limited our ability to determine temporal changes in microbial succession and cytokine dynamics. Second, investigating acute trauma inevitably involves complex clinical realities. Variables such as in-hospital antibiotic use, dietary support, and specific medications (e.g., proton pump inhibitors, analgesics) are known to influence the microbiome and represent a universal challenge in clinical TBI cohorts. Although we could not systematically control for all these factors or specific injury biomechanics (e.g., traffic-related injuries and falls), future studies incorporating these as covariates will further refine our understanding of neuroinflammatory responses. Although adjustment for age, sex, BMI, and education suggested that the injury-group effect persisted, residual confounding cannot be fully excluded. Additionally, functional pathway results were inferred from metagenomic data and were not validated by direct measurements of fecal SCFAs, bile acids, plasma metabolites, or inflammatory signaling activity. Finally, given the substantial inter-individual heterogeneity of the human microbiome, the microbiota–cytokine associations did not survive strict FDR correction. Therefore, these findings should be framed as exploratory and hypothesis-generating, providing a preliminary framework that requires validation in larger, pre-registered longitudinal cohorts.

## Data Availability

The raw metagenomic sequencing data have been deposited in the Genome Sequence Archive (GSA) database (https://ngdc.cncb.ac.cn/gsa) under accession number PRJCA068620.
